# Unraveling the etiology of primary amenorrhea: Insights from a tertiary care center in Peshawar, Pakistan

**DOI:** 10.12669/pjms.41.3.11199

**Published:** 2025-03

**Authors:** Ambareen Samad, Arzoo Gul Bangash, Talat Naz

**Affiliations:** 1 Dr. Ambareen Samad, MBBS, FCPS Assistant Professor, Department of Obstetrics & Gynaecology, Khyber Teaching Hospital, Peshawar, Pakistan; 2 Dr. Arzoo Gul Bangash, MBBS, FCPS Assistant Professor, Department of Obstetrics & Gynaecology, Khyber Teaching Hospital, Peshawar, Pakistan; 3 Dr. Talat Naz, MBBS, FCPS Professor, Department of Obstetrics & Gynaecology, Khyber Teaching Hospital, Peshawar, Pakistan

**Keywords:** Gonadal Dysgenesis, Imperforate hymen, Mullerian agenesis, Primary amenorrhea

## Abstract

**Background & Objectives:**

The onset of menstruation heralds the beginning of feminism and fecundity. Primary amenorrhea is a distressing symptom and is often associated with late diagnosis and appropriate management. Our study aimed to find the frequency of primary amenorrhea along with its causes and subsequent management by a multidisciplinary team.

**Methods:**

In our descriptive case series patients presenting with primary amenorrhea to the Department of Gynaecology, Khyber Teaching Hospital Peshawar from January 2018 to December 2022, were included. Gynecologist initially analyzed the cases based on history, clinical examination (secondary sexual characteristics, Body mass index) and relevant investigations including hormonal profile, pelvic ultrasound, MRI and karyotype. After final diagnosis, a multidisciplinary team comprising of plastic surgeon, endocrinologist and psychiatrist advised treatment based on cause. Relevant information was recorded on predesigned proforma. Data was analyzed using SPSS 23.

**Results:**

The frequency of primary amenorrhea was 0.3% out of 18,504 patients seen in Gynaecology clinic. Forty-six (82%) patients were in the age group of 14-20 years. The mean body mass index was 22.8±2.4. Mullerian agenesis was seen in 22(39%) of patients, 20(35.7%) had outflow tract abnormality which were treated by vaginoplasty or hymenectomy/ resection of vaginal septum depending on cause. Ten (17.8%) patients diagnosed with gonadal dysgenesis. Four(7.1%) patients with constitutionally delayed menarche had spontaneous onset of menstruation.

**Conclusions:**

Disorders related to Mullerian agenesis are the most common cause of primary amenorrhea. Timely diagnosis by attending gynecologist and subsequent multi-modal approach, including psychological counseling, hormonal therapy, and tailored surgical interventions like neovagina creation by vaginoplasty, hymenectomy and laparoscopic gonadectomy can ensure appropriate management of this distressing condition in young girls.

## INTRODUCTION

Amenorrhea is defined as the absence of menstruation. Primary amenorrhea is defined as the absence of menarche in a girl with absent secondary sexual characteristics by 14 years of age and by 16 years in the presence of secondary sexual characteristics.^1^ Puberty is the defining event in adolescent girls’ life with the onset of menstruation as a sign of feminism and fecundity Primary amenorrhea is associated with a profound impact on the physical, social and psychological well-being of the patient.^2^

The reported incidence of primary amenorrhea is 0.065% to 0.75%.^3^,^4^The defects leading to the absence of menstruation are compartmentalized, with defects either in the uterus, ovaries, pituitary, hypothalamus or in outflow tract. The diagnostic workup for the patient presenting with primary amenorrhea should be thorough and meticulous involving focused history, targeted examination, hormonal evaluation, appropriate utilization of imaging modalities (pelvic ultrasound, MRI) and cytogenetic studies where indicated. It has a devastating impact on the psychological well-being of the patient.^5^ There are many causes of primary amenorrhea including mullerian duct anomalies, gonadal dysgenesis, and constitutionally delayed puberty. Anatomic or mullerian anomalies and gonadal dysgenesis are the commonly reported causes worldwide.^6^

Primary amenorrhea being a rarer presentation, is usually referred to a tertiary care hospital for workup and management. Most anatomical causes have good treatment outcomes with restoration of regular menstrual cycles and fertility.^7^ Patients presenting with primary amenorrhea should be evaluated thoroughly because early diagnosis and timely intervention can prevent long-term health and social consequences. This research study will aid the growing body of evidence on etiology, and increase awareness amongst healthcare professionals and residents regarding early diagnosis and timely management of primary amenorrhea.

## METHODS

This descriptive case series study was done in the Gynaecology outpatient clinic of Khyber Teaching Hospital, Peshawar from January 2018 to December 2022. These patients were managed by a multidisciplinary team comprising a gynecologist, endocrinologist, plastic surgeon, and psychiatrist.

### Ethical Approval:

The study protocol was approved by the institutional research and ethical review board (IREB No.: 112/DME/KMC, dated January 9, 2018).

Pregnant women and those presenting with secondary amenorrhea were excluded. Informed consent was taken from the patient/guardian. In patients with primary amenorrhea, in presence of normal secondary sexual characteristics, pelvic examination was done to find outflow tract abnormalities, this was supplemented by pelvic ultrasound, MRI and diagnostic laparoscopy. Patients with absent secondary sexual characteristics were advised to do hormonal profile and karyotyping if needed. Information regarding relevant history, physical examination, including height, weight, tanner staging of secondary sexual characteristics like breast and pubic, axillary hairs, results of the hormonal profile (estradiol, luteinizing hormone, follicle-stimulating hormone, testosterone, thyroid function, and prolactin levels), pelvic ultrasound, MRI, laparoscopy and karyotyping findings for establishing the cause of primary amenorrhea, were recorded on a predesigned proforma. After the work-up patients were categorized into the following groups:

Group-1-Outflow tract abnormalities

Group-2-Mullerian agenesis

Group-3-Gonadal Dysgenesis

Group-4-Hypothalamic-pituitary disorders

Group-5-Delayed Menarche

Treatment either in the form of surgery, hormones or referral to an endocrinologist or plastic surgeon was recorded on predesigned proforma.

### Statistical Analysis:

Data was analyzed in SPSS package version 23. Continuous data was shown as the mean ± standard deviation, and the categorical and nominal data was presented as frequencies and percentages.

## RESULTS

In our study, there were 56 patients presented with primary amenorrhea. Forty-six (82%) of the patients were in the age group of 14-20 years. The mean Body Mass index was 22.8±2.4 Kg/m^2)^ . Forty-nine (87.5%) were unmarried and 7(12.5%) were married.

Seventeen (30%) patients had imperforate hymen. Mayer Rokistansky Kuster Hauser syndrome was found in 13(23%) while hypoplastic uterus and vaginal septum in 9(16%) and 3(5%) respectively. Turner syndrome was diagnosed in 5(9%), androgen sensitivity in 4(7%) and Swyer syndrome in 1(1.7%) of patients presenting with primary amenorrhea (Table-II and Table-III).

**Table-I:** Baseline characteristics of patients with Primary amenorrhea.

Variable		Value
Age	14-20	46 (82%)
	21-25	6 (11%)
	25-30	4 (7.1%)
Mean Body Mass Index (Kg/m^2)^		22.8±2.4
Family History of Primary Amenorrhea	Present	5 (9%)
	Not Present	51 (91%)
Marital status	Unmarried	49 (87.5%)
	Married	7(12.5%)

**Table-II:** Frequency of causes of primary amenorrhea.

Causes	No of Cases	Percentages
Mullarian Agenesis	22	39.2%
Outflow tract abnormalities	20	35.7%
Gonadal Dysgenesis	10	17.8%
Delayed Menarche	4	7.1%

**Table-III:** Clinical Profile of patients with Primary Amenorrhea.

Diagnosis	No	Sonographic/Laparoscopic findings		Karyotype	FSH/ LH	Treatment
		Uterus	Ovaries			
Imperforate Hymen	17	Normal	Normal	Not done	Not done	Surgical
MRKH	13	Absent/Rudimentry	Normal	46XX	Normal	vaginoplasty
Vaginal Septum	3	Hematometra	Normal	Not done	Normal	Surgical
Hypoplastic Uterus	9	Hypoplastic	Normal	Not done	Normal	Reassured
Androgen Insensitivity	4	Absent	Absent	46XY	Low	Referred
Swyer Syndrome	1	Normal	Streak	46XY	Normal	Referred
Turner Syndrome	5	Hypoplastic	Streak	45XX/XO	High	Hormonal
Delayed Menarche	4	Normal	Normal	Not done	Normal	Reassured

## DISCUSSION

In our study, the frequency of primary amenorrhea was 0.3% out of 18504 patients presenting at the Gynaecology outpatient department. This is somewhat similar to studies from interior Sindh, Karachi, and Quetta reporting frequency ranging from 0.2-0.7% depending on population and presence of tertiary care hospitals in particular area.^8^-^10^ Disorders related to Mullerian agenesis were seen in 22(39%) of cases with Mayor Rokitansky Kuster Hauser (MRKH) syndrome, which is described by primary amenorrhea, well-developed secondary sexual characteristics, female external genitalia, blind vagina, an absent or rudimentary uterus, and 46XX chromosomes, was seen in thirteen patients and hypoplastic uterus with normal vagina in nine patients. Five out of nine patients with hypoplastic uterus had a family history of delayed menarche in sisters as well suggesting a familial tendency that needs to be explored by further studies involving genetic testing.

Studies in Islamabad, India and Thailand also showed Mullerian agenesis as the commonest cause of primary amenorrhea.^11^,^12^ The Reported incidence of MRKH syndrome is one in 4500 with most of the cases being sporadic but genetic or familial tendency has also been observed similar to our study. Patients with MRKH syndrome were managed by a multidisciplinary team involving a gynecologist, plastic surgeon and psychiatrist to ensure their psychosocial and mental well-being. These patients underwent vaginoplasty to create neovagina for subsequent consummation of marriage later on.^13^-^15^ In our study, 17(30%) of girls had imperforate hymen, similar to figures reported by a study in Hyderabad and Lahore^16^. Three (5%) patients had transverse vaginal septum. All these abnormal outflow tract problems had excellent prognoses after hymenectomy or excision of vaginal septum, with restoration of menstruation and fertility.

Five (8.9%) girls in our study presented with primary amenorrhea, short stature and underdeveloped secondary sexual characteristics with a karyotype of 45XO. The multidisciplinary approach to exclude cardiac, renal and autoimmune disorders and hormone replacement therapy ( combined estrogen and progesterone) in these patients led to good outcomes in terms of onset of regular menstrual cycles later on. Four (7%) of patients presented with normal breast development and absent uterus, blind vagina, but relatively tall stature with a karyotype of 46 XY in two of them leading to a diagnosis of androgen insensitivity syndrome. One patient had normal mullerian structures and 46XY karyotype, a typical presentation of Swyer syndrome in which there is mutation and deletion in the SRY gene on the Y chromosome. These different variants of gonadal dysgenesis are similar to what is reported in worldwide studies although with varying frequency.^17^Global Literature shows a greater prevalence of gonadal dysfunction leading to primary amenorrhea in Western countries whereas outflow tract anomalies are more prevalent in Asian- African countries.^18^

In our study, Mullerian abnormalities mostly contributed to the etiology of primary amenorrhea in contrast to Western studies, probably due to differences in environmental, racial and genetic influences.^19^,^20^ A study by the largest tertiary care hospital in Peshawar showed Gonadal dysgenesis as the commonest cause of primary amenorrhea in 24% of cases possibly because of selection and referral bias. Patients with gonadal dysgenesis were treated by a multidisciplinary team for psychologic counselling, laparoscopic gonadectomy and hormonal treatment after sex of rearing is chosen by the patient. Four (7%) of our patients had normal stature and secondary sexual characteristics and normal hormonal profiles leading to a diagnosis of constitutionally delayed menarche similar to other reported studies.^20^,^21^ They were counselled and reassured with regular follow-ups showing a subsequent onset of menstruation without any treatment.

### Limitation:

Our study highlighted the frequency, and causes of a relatively rare disorder of primary amenorrhea in a tertiary care hospital of Peshawar, Pakistan. The limitation of our study is that it is a single center so the study population was somewhat affected by referral patterns and secondly, follow up was limited. Further multicentre data on etiology and management of primary amenorrhea are warranted especially exploring familial predisposition in cases of hypoplastic uterus.

## CONCLUSIONS

Disorders related to Mullerian agenesis are the most common cause of primary amenorrhea. Timely diagnosis by attending gynecologist and subsequent multi-modal approach, including psychological counseling, hormonal therapy, and tailored surgical interventions like neovagina creation by vaginoplasty, hymenectomy and laparoscopic gonadectomy can ensure appropriate management of this distressing condition in young girls.

### Authors’ Contribution:

**AGB and AS:** Conception, design of the study, acquisition, analysis, interpretation of data.

**TN:** Did literature search, Critical review

**AGB:** Critical analysis, Responsible for the integrity and accuracy of the manuscript.

All authors have read and approved the final version.
